# Mechanisms of mutant SOD1 induced mitochondrial toxicity in amyotrophic lateral sclerosis

**DOI:** 10.3389/fncel.2014.00126

**Published:** 2014-05-09

**Authors:** Piia Vehviläinen, Jari Koistinaho, Goldsteins Gundars

**Affiliations:** Department of Neurobiology, A.I. Virtanen Institute for Molecular Sciences, University of Eastern FinlandKuopio, Finland

**Keywords:** superoxide dismutase activity, oxidative stress, mitochondria, intermembrane space, misfolding

## Abstract

In amyotrophic lateral sclerosis (ALS), mitochondrial dysfunction is recognized as one of the key elements contributing to the pathology. Mitochondria are the major source of intracellular reactive oxygen species (ROS). Increased production of ROS as well as oxidative damage of proteins and lipids have been demonstrated in many models of ALS. Moreover, these changes were also observed in tissues of ALS patients indicative of important role for oxidative stress in the disease pathology. However, the origin of oxidative stress in ALS has remained unclear. ALS linked mutant Cu/Zn-superoxide dismutase 1 (SOD1) has been shown to significantly associate with mitochondria, especially in the spinal cord. In animal models, increased recruitment of mutant SOD1 (mutSOD1) to mitochondria appears already before the disease onset, suggestive of causative role for the manifestation of pathology. Recently, substantial *in vitro* and *in vivo* evidence has accumulated demonstrating that localization of mutSOD1 to the mitochondrial intermembrane space (IMS) inevitably leads to impairment of mitochondrial functions. However, the exact mechanisms of the selectivity and toxicity have remained obscure. Here we discuss the current knowledge on the role of mutSOD1 in mitochondrial dysfunction in ALS from the novel perspective emphasizing the misregulation of dismutase activity in IMS as a major mechanism for the toxicity.

## Introduction

Amyotrophic lateral sclerosis (ALS) is a fatal paralyzing disease characterized by the selective degeneration of motor neurons in the spinal cord, brain stem and motor cortex. The majority (∼90%) of ALS cases are sporadic whereas some 10% of patients have dominantly inherited familial ALS (FALS). However, the forms share clinical features. The suggested causative factors for the motor neuron degeneration include oxidative stress, neuroimmune reactions, protein aggregation (Boillée et al., 2006) and mitochondrial abnormalities such as disturbed calcium homeostasis (Jaiswal and Keller, 2009; De Vos et al., 2012) and dysfunctional axonal transport (De Vos et al., 2007). Nevertheless, the mechanism is yet to be identified. Mutations in several genes, including C9orf72, superoxide dismutase 1 (SOD1), TAR DNA binding protein (TARDBP), Fused in sarcoma (FUS), angiotensin, alsin, senataxin, and vesicle-associated membrane protein (VAPB) genes, cause FALS (Finsterer and Burgunder, 2014; Kiernan, 2014). Mutations in the C9orf72 gene are estimated to account for 35% and SOD1 mutations for 20% of FALS cases. The first gene linked to FALS was SOD1 (Rosen et al., 1993) and by today more than 150 FALS associated mutations in SOD1 have been identified. There are two types of mutant SOD1 (mutSOD1) proteins inducing clinically similar disease: those that show wild type like enzymatic activity and those with markedly reduced activity (Valentine et al., 2005). In addition, lack of SOD1 does not lead to development of ALS in mice (Reaume et al., 1996). Thus, the deleterious effect of mutSOD1 is thought to involve acquired toxic property possibly due to misfolding, aberrant enzymatic activity (Bruijn et al., 2004) or disturbance of cellular homeostasis by incorporating into the lipid bilayer membrane (Allen et al., 2012).

## The role of superoxide dismutation in mitochondrial hydrogen peroxide generation and oxidative stress

**Figure 1:**
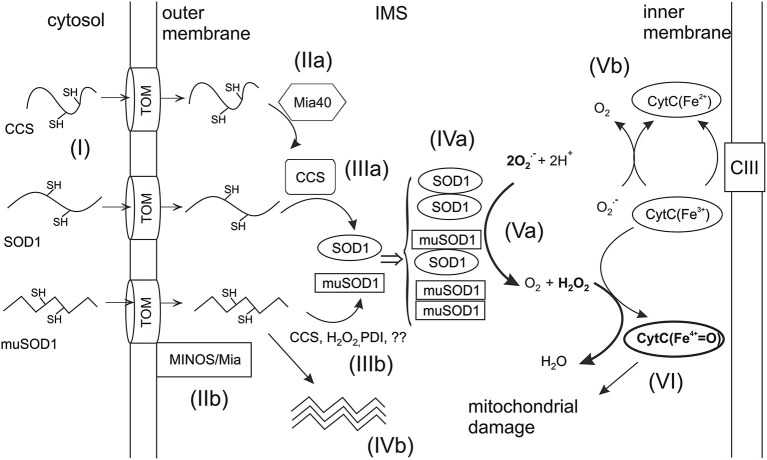
**Import of SOD1 and CCS into mitochondrial IMS**. Unfolded apoforms of SOD1 and CCS are imported into the IMS of mitochondria through the translocator of the outer membrane (TOM) (reaction I). In the IMS, apoCCS interacts with the import receptor, Mia40. Upon this interaction an intramolecular disulfide bond is formed within CCS trapping it in IMS (reaction IIa). The translocation of mutant SOD1 (muSOD1) may take place via alternative mechanism involving the mitochondrial inner membrane organization system (MINOS) and Mia40 (reaction IIb). Imported apoSOD1 interacts with mature CCS through a transient intermolecular disulfide bond (reaction IIIa), which promotes the formation of the intramolecular disulfide and subsequent trapping of SOD1 in IMS. Folding of muSOD1 can happen via interaction with CCS or by yet poorly known mechanisms such as oxidization by H_2_O_2_ or protein disulfide isomerase (PDI)—type of oxidoreductase (IIIb). Alternatively, mutants unable to fold correctly may form oligomers in IMS (reaction IVb). The disulfide oxidized mature monomers form the active enzyme by dimerization (reaction IVa). In ALS, mutant and wt homodimers may co-exist with heterodimers. Mature dimeric SOD1 dismutates superoxide (O_2-_) to O_2_ and H_2_O_2_ (reaction Va). Alternatively, superoxide may react with CytC^3+^ which oxidizes it to O_2_ (Vb). CytC^3+^ can also catalyze peroxidation in the presence of H_2_O_2_ yielding oxoferryl-cytC (CytCFe^4+^) which as a highly reactive oxidant may cause mitochondrial damage (reaction VI). The critical reactions (Va and VI) leading to mitochondrial damage are marked with bolded arrows.

Reactive oxygen species such (ROS) as superoxide and hydrogen peroxide (H_2_O_2_) are products of normal oxygen metabolism in cells (Dröge, 2002). They serve as signaling molecules but when present in excess can harm the structure and functions of the cell. In the cytoplasm, SOD1 dismutates superoxide anion radical to H_2_O_2_ that is further reduced to H_2_O by catalase, glutathione peroxidases or peroxiredoxins (Rhee et al., 2005). In intermembrane space (IMS), SOD1 has been suggested to play the similar protective role in handling of superoxide as in the cytosol (O’Brien et al., 2004; Aquilano et al., 2006; Klöppel et al., 2010; Fischer et al., 2011; Figure 1, reaction V). However, in this location, the scavenging systems might not be efficient enough to eliminate the H_2_O_2_ produced by dismutation. Upon cellular stress and pathological conditions such as ALS, the elevated H_2_O_2_ levels could contribute to mitochondrial damage.

Alternatively, superoxide in IMS could be detoxified by cytochrome C (cytC) which oxidizes it to O_2_ without producing secondary ROS (Figure 1, reaction Vb), thus providing a safe pathway to eliminate superoxide. However, cytC can also catalyze peroxidation in the presence of H_2_O_2_ yielding oxoferryl-cytC (CytCFe^4+^) which is a highly reactive oxidant (Figure 1, reaction VI). Indeed, we have demonstrated earlier a novel mechanism by which increased dismutase activity in IMS leads to cytC dependent peroxidation, followed by mitochondrial dysfunction and apoptosis (Goldsteins et al., 2008). Cells lacking SOD1 produced less H_2_O_2_ and were less sensitive to apoptosis induced by inhibition of mitochondrial respiratory complex III. Moreover, mitochondria isolated from spinal cords of G93A mice showed increased dismutase activity and elevated ROS production compared to wild type (wt) mitochondria. Further *in vivo* studies revealed that mutSOD1 in spinal cords of G93A SOD1 rodents is destabilized and bound to the inner mitochondrial membrane (Ahtoniemi et al., 2008). These results raise two essential issues for understanding the mechanisms of mutSOD1 induced mitochondrial toxicity: translocation of SOD1 to IMS and activity control of IMS localized SOD1.

## Factors regulating the import of SOD1 into mitochondria

A substantial proportion of SOD1 is found in the mitochondria (Weisiger and Fridovich, 1973; Jaarsma et al., 2001; Mattiazzi et al., 2002), concentrated in the IMS (Okado-Matsumoto and Fridovich, 2001; Sturtz et al., 2001). In mammalian cells, the mitochondrial localization of SOD1 is regulated by its folding as it can cross the mitochondrial membrane only in unfolded disulfide-reduced and non-metallated apo-form (Kawamata and Manfredi, 2008; Figure 1, reaction I). In addition, it was demonstrated that the subcellular localization of SOD1 is dictated by distribution of the copper chaperone for SOD1 (CCS; Kawamata and Manfredi, 2008). The maturation of SOD1 requires zinc binding followed by copper insertion and oxygen-dependent intramolecular disulfide bond formation (Furukawa et al., 2004), the last two actions being executed by CCS (Culotta et al., 1997). For proteins such as SOD1 and CCS that lack the mitochondrial localization signal, there is a disulfide relay system in IMS which drives their import (Mesecke et al., 2005). This Mia40/Erv1 system is involved in the translocation of yeast and mammalian SOD1 and CCS (Kawamata and Manfredi, 2008; Reddehase et al., 2009; Groß et al., 2011; Klöppel et al., 2011). When the reduced apo-proteins enter the IMS, the Mia40/Erv1 machinery introduces intramolecular disulfide bonds into them (Herrmann and Riemer, 2010). This folding step traps the proteins in the IMS as only unfolded precursors can use the translocator on the mitochondrial outer membrane (TOM) for exit.

ApoCCS is a direct substrate for Mia40 (Figure 1, reaction IIa) and the critical cysteines for their interaction in domain I of CCS were identified by Klöppel et al. (2011). The IMS-located CCS promotes folding of apoSOD1 and its subsequent trapping in IMS (Kawamata and Manfredi, 2008; Figure 1, reaction IIIa). High levels of CCS have been observed in the IMS of yeast and mammalian cells (Field et al., 2003; Kawamata and Manfredi, 2008) and CCS was found essential for mitochondrial localization of SOD1 in yeast (Field et al., 2003). Accordingly, the amount of mitochondrial SOD1 increases upon overexpression of CCS in mice (Son et al., 2007). In contrast, under the normal cell culture conditions overexpression of CCS resulted in decreased translocation of both CCS and SOD1 into the mitochondria (Kawamata and Manfredi, 2008). This appeared to happen because of the high oxygen concentration which promoted the oxidative folding of CCS and subsequent maturation of SOD1 in the cytosol. Lowering the O_2_ concentration from 20% to 6% that more resembles the conditions in tissues directed CCS and SOD1 to IMS (Kawamata and Manfredi, 2008). These data indicate tight O_2_-dependent physiological regulation of CCS and SOD1 localization.

Redox status of the cysteine residues in human SOD1 is critical for its retention in mitochondria due to their involvement in intramolecular disulfide bonds and in the interaction with CCS. There are four Cys residues in SOD1 (C6, C57, C111 and C146) which all have a role in mitochondrial import and accumulation (Kawamata and Manfredi, 2008). An intramolecular disulfide bridge is formed between C57 and C146 upon maturation (Arnesano et al., 2004) thus making these residues essential for the retention of SOD1 in IMS. C6 and C111 are not involved in intramolecular bonds but rather in intermolecular interactions (Niwa et al., 2007; Cozzolino et al., 2008). These interactions appear to be important as Cys-111 was identified as a key mediator of mitochondrial association *in vitro* (Ferri et al., 2006) and mutation in either of the two residues leads to poor accumulation of SOD1 in IMS (Kawamata and Manfredi, 2008).

A mitochondrial import mechanism for SOD1 independent of CCS has been described in yeast (Varabyova et al., 2013). This utilizes the mitochondrial inner membrane organization system (MINOS) and involves also Mia40. Interestingly, reduced SOD1 was retained in the mitochondria of CCS null cells in yeast (Varabyova et al., 2013). In mammalian cells, CCS independent mitochondrial import and retention exists for mutSOD1 (Kawamata and Manfredi, 2008).

## Control of SOD1 activity by its redox state, metal binding and quaternary structure

For maturing into an active enzyme, SOD1 requires three post-translational modifications: the insertions of zinc and copper, intramolecular disulfide bond formation, and dimerization. Zinc is deposited close to the active site stabilizing the structure (Potter et al., 2007). Copper is required for the enzymatic activity and is delivered to the catalytic center by assistance of CCS (Culotta et al., 1997). CCS also participates in the oxidation of the intramolecular disulfide bond between C57 and C146 in SOD1 (Furukawa et al., 2004) which results from disulfide transfer involving C244 and C246 in CCS (Banci et al., 2012). Finally, two metallated and disulfide-oxidized SOD1 monomers form a dimeric active enzyme via hydrophobic and hydrophilic interactions. The mature SOD1 is extremely stable due to the intramolecular disulfide bond which brings stability to the quaternary structure (Arnesano et al., 2004; Hörnberg et al., 2007).

Full activation of human SOD1 requires CCS (Wong et al., 2000) but an activation pathway independent of CCS also exists (Carroll et al., 2004). This alternative way of activation requires reduced glutathione but is independent of oxygen (Carroll et al., 2004; Leitch et al., 2009). Thus, in contrast to oxygen-dependent CCS-mediated activation this pathway allows SOD1 activation in low oxygen conditions. Notably, in tissues of mice null for CCS as much as 10–20% of SOD1 activity has been observed compared to wt mice (Wong et al., 2000; Subramaniam et al., 2002).

## Aberrant mitochondrial import and activity control of SOD1 in ALS

### Accumulation of mutSOD1 in mitochondria

In ALS, mutSOD1 is recruited to mitochondria especially in the spinal cord (Higgins et al., 2002; Liu et al., 2004). Oxygen concentration critically influences cellular distribution of CCS and subsequently that of wtSOD1 whereas mutSOD1 appears to be able to escape this physiological regulation leading to mitochondrial accumulation of mutants also in the absence of CCS (Kawamata and Manfredi, 2008). Recently, it was demonstrated that reduced mutSOD1 enter the mitochondria via CCS-independent route involving MINOS and Mia40 (Varabyova et al., 2013; Figure 1, reaction IIIb). In accordance with the implications of the redox status of Cys residues in human SOD1 for its mitochondrial association, a variety of SOD1 mutants have been observed to possess susceptibility to the reduction of disulfide bonds *in vitro* (Tiwari and Hayward, 2003). These findings are in agreement with studies implicating the presence of reduced SOD1 in spinal cords of ALS mice (Jonsson et al., 2006; Karch et al., 2009; Zetterström et al., 2013). Even though the *in vivo* studies did not assess the localization of reduced mutSOD1, one could expect that it would be accessible to mitochondria where a part of that pool could be oxidized. In cell culture, oxidation of Cys111 residues led to accumulation of several SOD1 mutants in mitochondria (Ferri et al., 2010). However, another study demonstrated that, in contrast to wtSOD1, mitochondrial association of mutSOD1 does not depend on Cys111 (Kawamata and Manfredi, 2008).

Furukawa and O’Halloran (2005) suggested that the reduction of conserved disulfide bond predisposes SOD1 to incorrect crosslinking and aggregation (Figure 1, reaction IVb). Certainly, formation of intracellular aggregates has caught much attention as it is a hallmark of ALS. Indeed, several mouse models show correlation between insoluble aggregated forms of mutSOD1 and manifestation of symptoms (Johnston et al., 2000; Wang et al., 2002, 2003) in concert with findings from human tissues (Kato, 2008). Moreover, mutants possessing a higher aggregation propensity correlated with shorter disease duration in patients when 33 different SOD1 mutants were studied (Prudencio et al., 2009). In contrast to that correlation, D101N, I113T and L144F mutations with low or modest propensity to aggregate provoked rapidly progressing disease (Ayers et al., 2014). Despite of intensive investigations the mechanism how aggregates would result in toxicity has remained unclear. It has been suggested that soluble forms of mutSOD1 initiate the disease (Zetterström et al., 2007; Karch et al., 2009) and are, in fact, more toxic than insoluble aggregates (Zetterström et al., 2007; Brotherton et al., 2012; Weichert et al., 2014). Thus, the key appears to involve misfolding and the localization in the mitochondria rather than aggregate formation. Soluble misfolded subfractions of mutSOD1 were found enriched in spinal cords of ALS mice (Zetterström et al., 2007), and the conformation of such species could facilitate mitochondrial translocation. Investigators utilizing antibodies selectively recognizing misfolded/non-native SOD1 have demonstrated association of misfolded SOD1 with spinal cord mitochondria in mutSOD1 rodents (Velde et al., 2008; Brotherton et al., 2012). In cultured motor neuronal cells, obligate expression of mutSOD1 in IMS leads to mitochondrial toxicity and cell death (Cozzolino et al., 2009; Magrané et al., 2009). The toxicity was abolished by overexpression of glutaredoxin 2 possibly via modulation of disulfide bonds or by affecting mitochondrial redox environment (Ferri et al., 2010).

Most forms of mutSOD1 show instability *in vitro* (Rodriguez et al., 2002, 2005; Lindberg et al., 2005) and mitochondrial mutSOD1 has been reported to exist in partially unfolded oligomerized state (Deng et al., 2006; Ferri et al., 2006). Interestingly, Synofzik et al. (2012) reported on patients carrying a novel SOD1 mutation, L117V, which was indistinguishable from wtSOD1 in terms of stability and dismutase activity resulting in slowly progressing form of ALS. These findings are consistent with earlier data demonstrating that even small amounts of misfolded mutSOD1 are enough to induce ALS (Jonsson et al., 2006). In addition, overexpressed wtSOD1 is also able to acquire an unstable, misfolded conformation, leading to motor neuron disease in mice (Graffmo et al., 2013).

### The role of dismutase activity in mutSOD1 induced mitochondrial toxicity

The IMS-located SOD1 has been suggested to be inactive in the intact mitochondria and activated through oxidative modification of its critical thiol groups by yet poorly known mechanisms (Iñarrea et al., 2005). Strikingly, overexpression of CCS in G93A-SOD1 mice results in enhanced mitochondrial recruitment of SOD1 and drastically accelerates disease progression (Son et al., 2007). Further, the worsened outcome was independent of protein aggregation, leaving the possibility of the involvement of aberrant dismutase activity. Indeed, increased dismutase activity was found in mitochondria isolated from spinal cords of G93A mice compared to wt mitochondria and it was accompanied with elevated ROS production (Goldsteins et al., 2008). Accordingly, mitochondrial state 4 respiration was increased in spinal cord and brain mitochondria of presymptomatic G93A rats and it was coupled with increased production of hydrogen peroxide (Panov et al., 2011). This is in agreement with observed hypermetabolism in ALS patients (Bouteloup et al., 2009). In addition, recent data on pulmonary fibrosis shows that translocation of SOD1 to IMS leads to increased production of H_2_O_2_ (He et al., 2011).

Insertion of copper is required for the activity of SOD1 and altered copper homeostasis is a common feature in mutSOD1 mice (Tokuda et al., 2013). Cu(atsm) treatment prolongs the survival of ALS mice (Soon et al., 2011). Importantly, in the spinal cords of those mice the cytoplasmic dismutase activity was increased upon copper supplementation. Even though the mitochondrial activity was not assessed one could hypothesize that it could be decreased: if more SOD1 is activated in the cytoplasm then the pool of apo-form capable of entering mitochondria should decrease. When mutSOD1 mice were treated with copper chelator the onset of disease was delayed and the mice survived longer (Hottinger et al., 1997; Tokuda et al., 2008). This improvement was accompanied with decreased dismutase activity in spinal cord homogenates (Tokuda et al., 2008). In cell culture, copper depletion markedly increased the viability of G93A motoneurons (Azzouz et al., 2000). In another study, copper depletion has been shown to increase mitochondrial association of SOD1 (Arciello et al., 2011). In the latter, the cell viability was not assessed and the mitochondrial morphology was not altered. It is possible that in the above mentioned studies on copper depletion, the mitochondrial import of SOD1 was increased but due to the lack of copper the enzyme remained inactive and thus unable to cause mitochondrial damage. However, the lack of CCS, which provides SOD1 with copper, does not rescue mice from mutSOD1 induced ALS (Subramaniam et al., 2002). Moreover, when all four histidine residues that coordinate copper binding are knocked out from mutSOD1 mice they still develop motor neuron disease (Wang et al., 2003). These latter two findings could be explained by alternative ways of SOD1 activation and by heterodimer formation (Witan et al., 2008, 2009).

In addition to proper folding and metal acquisition, dimerization is critical for dismutase activity. Mutant and wtSOD1 are able to form either homo- or heterodimers both in animal and cell culture models (Furukawa et al., 2006; Witan et al., 2008; Wang et al., 2009). Interestingly, wtSOD1 monomer is able to provide enzymatic activity to the heterodimer where the other monomer is an inactive mutant (Witan et al., 2008). Furthermore, it was found that the toxicity of mutSOD1 does not correlate with its aggregation potential but with the ability to form active dimeric molecules (Witan et al., 2008). There is some discrepancy in current data published on the role of wtSOD1 in FALS. However, most of the rodent models of ALS where wtSOD1 is co-expressed with mutSOD1 show earlier onset of the disease (Jaarsma et al., 2000; Fukada et al., 2001; Deng et al., 2006; Wang et al., 2009). Notably, in the work by Wang et al. (2009) the phenotype was associated with markedly increased dismutase activity in spinal cord extracts. Both Furukawa and Wang also confirmed the existence of heterodimers *in vivo*, suggesting that formation of heterodimers may be physiologically relevant in FALS patients as well. It has been proposed that the heterodimers comprise a pool of more soluble and more toxic SOD1 whereas homodimers form less toxic aggregates (Weichert et al., 2014). Furthermore, it was demonstrated that wtSOD1 actually reduces aggregate formation of mutSOD1 in cell culture (Witan et al., 2009). How different dimers affect mitochondrial dismutase activity and H_2_O_2_ production is not known and warrants further elucidation. In the model proposed (Figure 1, reaction IV) enzymatically active homo- and heterodimers all contribute to dismutation of superoxide. Accumulation of these dimers leads to increased activity which is likely augmented by loss of physiological controlling mechanism. Subsequent increase in H_2_O_2_ leads to cytC catalyzed peroxidation and mitochondrial damage.

## Conclusions

Under physiological conditions SOD activity in IMS is suppressed by redox state control. However, upon mitochondrial stress SOD1 may undergo oxidative activation and compete with cytochrome C for superoxide released in the IMS, leading to increased ROS production. In ALS, mutSOD1 accumulates in IMS, thus contributing to the misregulation of dismutase activity. The presence of endogenous wt enzyme may provide dismutase activity to inactive mutant by heterodimerization. Here we propose that aberrant regulation of dismutase activity in IMS as a major mechanism for the toxicity should be further evaluated in future studies. Furthermore, the possible involvement of mitochondrial IMS dismutase activity misregulation in sporadic ALS warrants careful assessment.

## Conflict of interest statement

The authors declare that the research was conducted in the absence of any commercial or financial relationships that could be construed as a potential conflict of interest.
